# Supplementary material for: Deletion of pbpC Enhances Bacterial Pathogenicity on Tomato by Affecting Biofilm Formation, Exopolysaccharides Production, and Exoenzyme Activities in Clavibacter michiganensis
Source: Int J Mol Sci. 2023 Mar 10;24(6):5324. doi: 10.3390/ijms24065324 (PMC10049144; doi:10.3390/ijms24065324)
Supplement: Supplementary file 1 [file ijms-24-05324-s001.zip › ijms-2061541-supplementary.pdf]

International Journal of *Molecular Sciences*

Supporting information legends

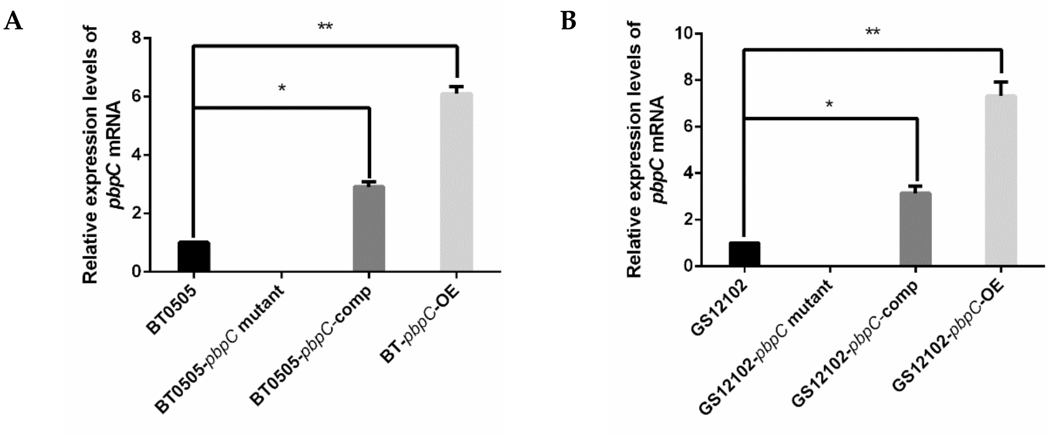

Figure S1. Expression levels of *pbpC* determined by qRT-PCR for both wild type strains BT0505 and GS12102 compared with *△pbpC* mutants, *pbpC*-comp, and *pbpC*-OE strains. Relative gene expressions were normalized with *gyrB*, *gapA*, and *bipA* which we employed as housekeeping references. Results presented as means of three independent experiments with standard deviation (n=3).

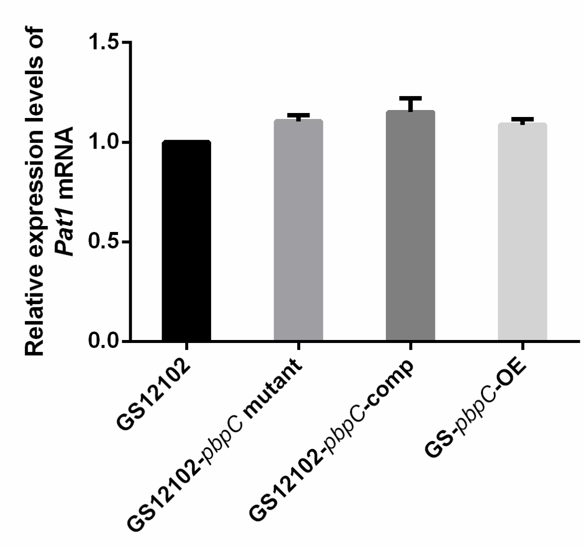

Figure S2. Transcript levels of *pat-1* located on plasmid pCM2 determined by qRT-PCR for both wild-type strains BT0505 and GS12102 compared with *△pbpC* mutants, *pbpC*-comp, and *pbpC*-OE strains. Relative expression of *pat-1* was only detectable in strain GS12102 but showed no meaningful C_t value in strain BT0505, as expected. Relative gene expressions were normalized with *gyrB*, *gapA* and *bipA*, which we employed as housekeeping references. Values are the means of three independent experiments with standard deviation (n=3).

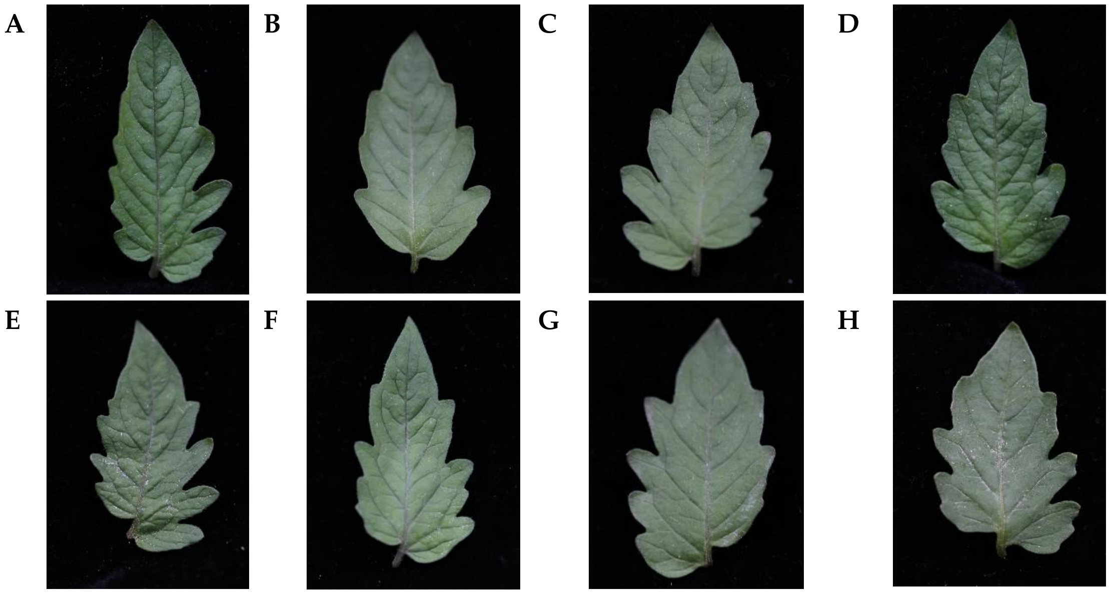

Figure S3. Pathogenicity test, in vitro, was performed by spraying gradient EPS solution onto freshly harvested *S. lycopersicum* leaves placed in Petri dish with moist sterile gauze at 4°C for 7 days. (**A-H**) *S. lycopersicum* leaves sprayed with water solution control, 5 mg/mL sucrose solution, or 0.01, 0.1, 0.5, 1, 2, or 5 mg/mL EPS solution, respectively, were photographed after 7 days of spraying procedure.

International Journal of Molecular Sciences

Table S1. Bacterial strains and plasmids used in the study.

Strain/Plasmid	Description	Source
Strains		
BT0505	Wild type strain harbors only pCM1 plasmid	Lab-preserved strain
GS12102	Wild type strain harbors both pCM1 and pCM2 plasmids	Lab-preserved strain
BT0505-*pbpC* mutant (*ΔpbpC*)	Knockout mutant for *pbpC* from parental BT0505 strain obtained by homologous recombination using plasmid pEB-C, Chl^R	Chen et. Al., 2021
GS12102-*pbpC* mutant (*ΔpbpC*)	Knockout mutant for *pbpC* from parental GS12102 strain obtained by homologous recombination using plasmid Cm0915-C, Chl^R	Current study
BT0505-*pbpC*-comp (*ΔpbpC*-comp)	Complementation strain obtained by transformation of BT0505-*pbpC* mutant by plasmid pHN-C, Chl^R, Neo^R	Chen et. Al., 2021
GS12102-*pbpC*-comp (*ΔpbpC*-comp)	Complementation strain obtained by transformation of GS12102-*pbpC* mutant by plasmid pHN-GS-C, Chl^R, Neo^R	Current study
*E. coli Trelief*5α	Chemically competent cell	Tsingke biological technology, Beijing, China
Plasmids		
pEASY-T1 Vector	Simple Cloning Vector, Km^R	TransGen Biotech, Beijing, China
pOKU9cmB*α*	Donor plasmid for *cmx* selectable marker cassette encoding chloramphenicol resistance.	(Kaup et al., 2005) [74]
pHN216	*E.coli-Clavibacter* shuttle vector, Neo^R	(Laine et al., 1996) [69]
pEB-C	Knockout plasmid of *pbpC* for parental BT0505 strain. *pEASY*-T1 vector derivative, Km^R Chl^R	Current study
Cm0915-C	Knockout plasmid of *pbpC* for parental GS12102 strain. *pEASY*-T1 vector derivative, Km^R Chl^R	Current study
pHN-C	Complementation plasmid of *pbpC* for parental BT0505 strain. pHN-216 derivate, Neo^R	Current study
pHN-GS-C	Complementation plasmid of *pbpC* for parental GS12102 strain. pHN-216 derivate, Neo^R	Current study

Abbreviation: Chl, chloramphenicol; Neo, neomycin; Km, kanamycin; R, resistance

International Journal of Molecular Sciences

Table S2. Primers used in qRT-PCR analysis in the our study.

Gene	Primer	Sequence (5′→3′)	Amplicon length (bp)
gyrB	gyrBRT-F1	GGACAGCACATCACGACCC	202
	gyrBRT-R1	CCTTCGGCATCTTCTTCCC	
bipA	bipART-F1	GGGTGCTGGTCGTCGTA	63
	bipART-R1	CGAGCCGCTGTTCAAG	
gapA	gapART-F1	TTGACCTGGTTGCCGATGAC	64
	gapART-R1	TCAACGACCCGCACTCCTC	
celA	celART-F1	GGTTCTCCGCATCAAACTATCC	85
	celART-R1	TGCTTGTCGCTCGTCGTC	
celB	celBRT-F1	GGAGACCACCAGCGACAAG	85
	celBRT-R1	TGAACGACCAGAACGACGAG	
pat-1	pat1RT-F1	GCTGATTCGCGAGAGGATC	85
	pat1RT-R1	GTTCTCGGTTGCTGTGTCGC	
chpC	chpCRT-F1	GACTGCTAATCACTGTGTTG	85
	chpCRT-R1	CAATAAACCGTTCCGATGG	
ppaA	ppaART-F1	AATCGGGCTGGTTCTGGTTT	85
	ppaART-R1	AGATTCTGCGGCATCTGCAT	
pelA	pelART-F1	GTGCGTTCCTGCGGTAAC	85
	pelART-R1	GCGGATGGTGATGTGGTC	
xysA	xysART-F1	ACGGGCAACAGCGAGAAC	85
	xysART-R1	GATCATCGTGAACAGGTCCTTG	
xysB	xysBRT-F1	AAGCCCGAGAGCGTCCAG	85
	xysBRT-R1	TGCGAGTGCCAGAACAGC	
pbpC	pbpCRT-F1	GGCTGAAGAGCGGGAAGT	136
	pbpCRT-R1	CCGGTGTTGGTGTCGTTG	

References

Kaup, O.; Gräfen, I.; Zellermann, E.-M.; Eichenlaub, R.; Gartemann, K.-H. Identification of a tomatinase in the tomato-pathogenic actinomycete *Clavibacter michiganensis* subsp. *michiganensis* NCPPB382. *Molecular plant-microbe interactions* **2005**, *18*, 1090-1098

Laine, M.J.; Nakhei, H.; Dreier, J.; Lehtilä, K.; Meletzus, D.; Eichenlaub, R.; Metzler, M.C. Stable transformation of the gram-positive phytopathogenic bacterium *Clavibacter michiganensis* subsp. *sepedonicus* with several cloning vectors. *Applied and environmental microbiology* **1996**, *62*, 1500-1506
